# Distribution and Characterization of Typical Antibiotics in Water Bodies of the Yellow River Estuary and Their Ecological Risks

**DOI:** 10.3390/toxics11050400

**Published:** 2023-04-23

**Authors:** Jindong Wang, Zhenfei Yan, Yu Qiao, Daqing Liu, Chenglian Feng, Yingchen Bai

**Affiliations:** 1 State Key Laboratory of Environmental Criteria and Risk Assessment, Chinese Research Academy of Environmental Sciences, Beijing 100012, China; 2 College of Water Science, Beijing Normal University, No. 19, Outer Street, Xinjiekou, Beijing 100875, China

**Keywords:** antibiotics, Yellow River Estuary, ecological risk assessment

## Abstract

A total of 34 antibiotics from five major classes of antibiotics, including macrolides, sulfonamides, quinolones, tetracyclines and chloramphenicol, were considered as contaminants, considering the Yellow River Estuary as the study area. The distribution, sources and ecological risks of typical antibiotics in the Yellow River Estuary were investigated using an optimized solid-phase extraction pre-treatment and an Agilent 6410B tandem triple-quadrupole liquid chromatography–mass spectrometer for antibiotic detection. The results show that antibiotics were widely present in the water bodies of the Yellow River Estuary, with 14 antibiotics detected to varying degrees, including a high detection rate for lincomycin hydrochloride. Farming wastewater and domestic sewage were the primary sources of antibiotics in the Yellow River Estuary. The distribution characteristics of antibiotics in the study area were linked to the development of farming and social activities. The ecological risk evaluation of 14 antibiotics in the Yellow River Estuary watershed showed that clarithromycin and doxycycline hydrochloride were present at medium-risk levels, and lincomycin hydrochloride, sulfamethoxazole, methomyl, oxifloxacin, enrofloxacin, sulfadiazine, roxithromycin, sulfapyridine, sulfadiazine and ciprofloxacin were present at low-risk levels in the samples collected from water bodies of the Yellow River Estuary. This study provides novel, beneficial information for the assessment of the ecological risk presented by antibiotics in the Yellow River Estuary water bodies and provides a scientific basis for future antibiotic pollution control in the Yellow River Basin.

## 1. Introduction

The Yellow River is the mother river of the Chinese nation. As the second largest river in China, it plays an essential role as the country’s northern drinking water supply and feeds the agricultural system, but it also receives natural or treated effluent from urban centers. A large amount of wastewater (up to 4.4 billion tonnes/year) is generated from industrial production, livestock farming and agricultural surface sources. Introducing effluent from multiple sources has led to a deterioration of the Yellow River’s water quality in localized sections, with large amounts of antibiotics detected frequently. In the 18th Party Congress, a national strategy was formed to promote the ecological protection and high quality of water in the Yellow River Basin [1]. In 2019, the proportion of Class I-II surface water quality sections in the Yellow River Basin was on the rise, and the balance between IV and poor V categories of water quality was on the decline. However, poor V sections still account for 8.8%, and water pollution in tributaries is still relatively severe [2]. In October 2021, the Ministry of Ecology and Environment issued the “Action Plan for the Treatment of New Pollutants (Draft for Public Comments)”, proposing specific targets and visions for the treatment of new pollutants [3]. In September 2022, the General Office of the Ministry of Ecology and the Environment issued the “List of New Pollutants for Priority Control (2022 Version) (Draft for Public Comments)”, proposing that antibiotic residues should be managed following hazardous waste protocols, and that the battle against pollution should be fought head on [4].

Antibiotics are organic substances synthesized naturally by microorganisms through secondary metabolism or synthesized artificially by industry. They can inhibit the growth or metabolic activity of other microorganisms and can even cause their metabolism and death [5,6]. As a new contaminant, antibiotics are used by humans and animals. Furthermore, due to incomplete intestinal absorption and incomplete metabolism, antibiotics can be excreted through feces and urine and enter water bodies [7], soil [8] and sediments [9]. Antibiotics have played an essential role in the development of modern medicine. Still, with their extensive clinical use, over 50,000 tonnes of antibiotic residues are “released” into the water environment each year [10], making many rivers major reservoirs of these pollutants [11,12]. Antibiotic contamination not only poses a severe risk to aquatic ecosystems [13,14], but can also induce microbial resistance, posing a severe threat to public health [15].

According to statistics, in 1999, 65% of the 13,216 tons of antibiotics used in Germany were applied to treat human diseases, and in Denmark, 20.8%, 27.4% and 51.8% were used for human, veterinary and growth purposes. The annual use of antibiotics in the United States is stipulated as 22,700 tons, 50% for humans and 50% for animals, agriculture and aquaculture [16]. China produces 75% of the total antibiotics, and the abuse of antibiotics is a serious situation. In recent years, most studies on antibiotic pollution in the Yellow River basin have focused on the Jinan section of the lower Yellow River [17], the Yellow River delta wetlands [18,19] and the lower Yellow River [20], and relevant studies on the Yellow River Estuary basin are still insufficient.

In the present study, typical antibiotics were detected in the surface waters of the Yellow River Estuary, and the distribution and characterization of antibiotics were also analyzed. At the same time, the ecological risks of antibiotics were assessed via the use of risk quotient methods (RQs). This study deepens the understanding of the concentration levels of antibiotics in the water bodies of the Yellow River Estuary, provides theoretical support for environmental protection of the water in the basin and provides a reference for maintaining the health of the ecosystem and drinking water safety in the Yellow River Estuary.

## 2. Materials and Methods

### 2.1. Sample Collection

**Figure 1:**
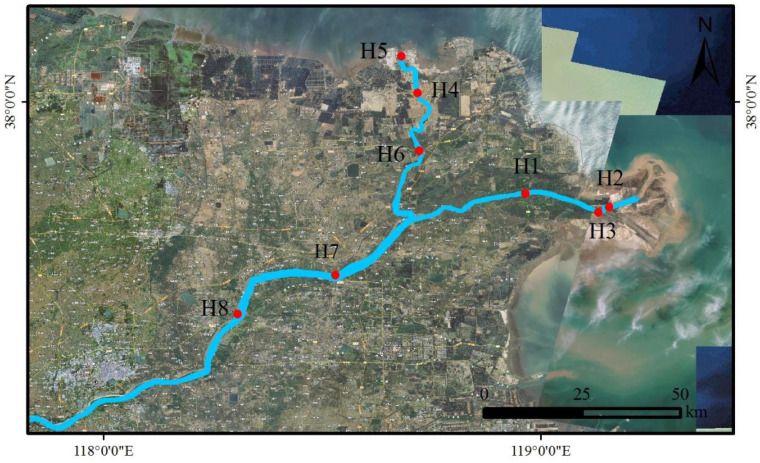
Sample points in the study area of the Yellow River.

The Yellow River Estuary is located in Dongying, Shandong Province, which is close to Bohai Bay and Laizhou Bay. It forms the fan-shaped accumulation plain of the modern-day Yellow River Estuary. The terrain is low in elevation with an average altitude below 15 m, and the range is between 37°34′–37°53′ N and 118°53′–119°21′ E (Figure 1). It has a temperate continental monsoon climate, four distinct seasons, an average yearly temperature of 12.1 °C, an average yearly precipitation of 530–630 mm with concurrent high rain and humidity.

Combined with field investigation and a review of relevant data, eight water samples were collected at eight points in the Yellow River Estuary in September 2022, as shown in Figure 1.

A total of 2 L of water was collected in a brown glass bottle, and the growth of bacteria in the water sample was inhibited by adding 50 mL of methanol to slow down the degradation of antibiotics by microorganisms. To improve antibiotic recovery, 100 μL of concentrated H_2_SO_4_ was added to the water sample, and the pH was adjusted to around 3. The sample was stored at a low temperature and transported back to the laboratory, where the samples were processed within 24 h.

### 2.2. Advanced Analysis Instruments and Reagents

An Agilent 6410B triple-quadrupole liquid chromatography–mass spectrometer (1290-6460, Agilent Technologies, USA); Extend-C18 Column (2.1 mm × 100 mm × 3.5 μm, Agilent Technologies, USA); Vortex (UVS-3, Beijing Yousheng United Technology Co., Ltd., China); electronic balance (AR224CN, OHAUS Instruments (Changzhou Co., Ltd., China); and CNC ultrasonic cleaner (KQ-250DE, Kunshan Ultrasonic Instrument Co., Ltd., China) were the main advanced analysis instruments employed.

A total of 34 antibiotic standards were grouped into five categories, i.e., (1) macrolides, including erythromycin, roxithromycin, hythromycin, azithromycin and tylosycin, clindamycin; (2) sulfonamides, including sulfaacetic amide, sulfaclodazine, sulfadimethoxypyrimidine, sulfapyridine, sulfathiazole, sulfamethiodiazole, sulfadiazine, sulfamethazine, sulfamethoxazole, sulfadimethazole, sulfadimethylpyrimidine, trimethoprim and sulfaquinoxaline; (3) quinolones, including ofloxacin, norfloxacin, ciprofloxacin, enrofloxacin, salafloxacin, lomefloxacin, flurofloxacin and difloxacin; (4) tetracyclines, including doxycycline hydrochloride, tetracycline (hydrochloride), oxytetracycline (oxytetracycline) and chlortetracycline (chlortetracycline); (5) chloramphenicol, including chloramphenicol, florfenicol, thiamphenicol and rifampicin. The above reagents were imported from the German company Dr. Ehrenstorfer.

Four internal standards, sulfamerazine-D4 (SMR-D4), ciprofloxacin-D8 (CIPROFLOXACIN-D8, CIP-D8), normeclocycline (DTC) and erythromycin-^13^C, D3 (erythromycin-^13^C, D3, ERY-^13^C, D3), were imported from Dr. Ehrenstorfer in Germany.

A Water Oasis HLB (6 mL, 200 mg) solid-phase extraction cartridge, methanol, acetonitrile, hydrochloric acid, Na_2_EDTA, ethyl acetate, dichloromethane, ammonium acetate, formic acid, disodium hydrogen phosphate and citric acid were purchased from Shanghai Anpu Experimental Technology Co., Ltd., and a 0.7μm (70 mm) GF/F filter membrane was purchased from Whatman Company in the United Kingdom.

### 2.3. Sample Treatment

The water sample was filtered through a 0.45 μm pore glass-fiber membrane, and weighed 1.0 L of water accurately. Eight samples were taken in two replicates for a total of sixteen samples. An amount of 0.2 g of Na_2_EDTA was added to reduce the chelation of antibiotics and metal ions in the water sample, about 300 μL of hydrochloric acid was added to the water sample to adjust the pH of the water sample to 3.0~4.0, 25 ng of antibiotic internal standard was added, and then the cartridge was extracted using solid-phase Oasis HLB (200 mg/6 cc) at a rate of 5 mL/min. The HLB cartridge was activated with 10 mL of methanol, 10 mL of purified water and 10 mL of pure water with a pH of 4.0. After the sampling, the column was cleaned with 10 mL of pure water, drained, dried under the protection of nitrogen for 30 min, eluted in 3 times using 6 mL of methanol, nitrogen-blown until nearly dry and reconstituted with the initial mobile phase (0.1% formic acid–ammonium formate aqueous solution/acetonitrile) to be measured.

### 2.4. Instrumental Analysis

The HPLC-MS/MS used the Agilent 6410B tandem triple-quadrupole LC-MS/MS, Waters Xterra C18 separation column (100 mm × 2.1 mm, 3.5 μm) ESI ionization source. Mobile phase: A phase, 0.1% formic acid–ammonium formate; B: acetonitrile. Linear gradient: 0 min, 5% B; 0.1~10 min, 10~60% B; 10~12 min, 60%; 12.1~22 min 10% B. The flow rate was 0.25 mL/min. The column temperature was maintained at 25 °C and the injection volume was 200 μL. MS conditions: gas temperature of 350 °C, gas flow rate of 8 mL, nebulizer pressure of 25 psi, capillary voltage of 4000 V.

### 2.5. Ecological Risk Assessment

Risk quotient methods (*RQs*) are one of the most effective methods for assessing the environmental risks of aquatic biochemicals [21]. This study used data on the antibiotic concentrations in the water of the Yellow River Estuary for ecological risk assessment. According to the methodology for environmental risk assessment presented in the EU’s technical guidance document, ecological risks can be assessed using the risk quotient (*RQ*).

The *RQ* is calculated as follows:

(1) $$RQ=\frac{MEC}{PNEC}$$

(2) $$PNEC=\frac{EC_{50}\left(LC_{50}\right)}{AF}$$

where *MEC* represents the measured environmental concentration and *PNEC* indicates the predicted non-effect concentration for each contaminant. *PNEC* is the quotient of the toxicological relevant concentration with an appropriate assessment factor (*AF*). *LC*_50_ represents the median lethal concentration and *EC*_50_ represents the half maximal effective concentration. *LC*_50_ or *EC*_50_ represent the lowest maximal effective concentration value according to the available literature. According to the RQ, it can be divided into three risk levels: high risk (*RQ* > 1), medium risk (0.1 < *RQ* < 1) and low risk (*RQ* < 0.1) [22].

### 2.6. Data Analysis

The sampling sites were mapped using ArcGIS software and Bigemap Gis Office software. OriginPro 2023 software was also used to produce box plots and bar charts to visualize and clearly show the distribution of antibiotics in the Yellow River Estuary waters at the eight sampling sites.

## 3. Results and Discussion

### 3.1. Concentration Levels of Antibiotics in the Water Bodies of the Yellow River Estuary

**Figure 2:**
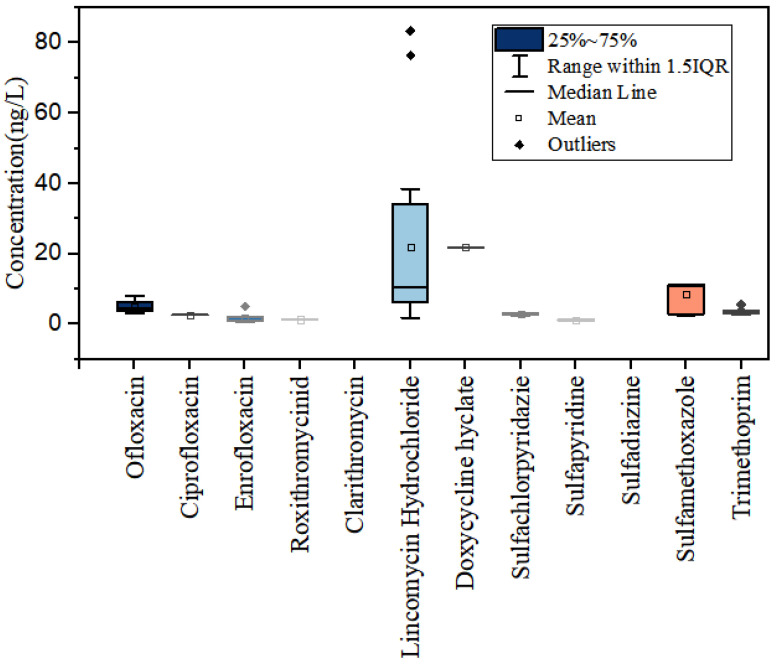
Box plots of measured concentrations of twelve antibiotics in water samples from the Yellow River Estuary.

The results of the antibiotic monitoring experiment in the Yellow River Estuary are shown in Figure 2, with the maximum, 75th percentile, mean, median, 25th percentile and minimum values shown in order from highest to lowest in the box plot.

Sulfonamides had the highest average concentration in the waters of the Yellow River Estuary, including all sulfonamide derivatives and sulfa analogs based on the chemical synthesis of p-aminobenzenesulfonamide, whose structures are connected to a free amino and sulfonamide group in the para-position of the benzene ring. These are broad-spectrum synthetic antibacterial agents with the advantages of low price, stable performance and good therapeutic effect. They are commonly used in the medical, agricultural, aquaculture and livestock industries for the prevention and treatment of bacterial and protozoan infections [23]. Their average concentration in the water bodies of the Yellow River Estuary reached 11.80 ng·L^−1^; this was followed by macrolides at 8.40 ng·L^−1^, quinolones at 4.40 ng·L^−1^, tetracyclines at 1.37 ng·L^−1^ and chloramphenicol at the lowest level, that is, not detected. The highest concentration detected at any site was 83.31 ng·L^−1^, found at sampling site H2. As a macrolide, lincomycin hydrochloride has similar effects to erythromycin and has a better impact on Gram-positive cocci. The antibiotics with the highest mean concentrations seen at each site were, in descending order, lincomycin hydrochloride (8.36 ng·L^−1^), sulfamethoxazole (8.91 ng·L^−1^), ofloxacin (5.08 ng·L^−1^), methicillin (4.27 ng·L^−1^), sulfamonomethoxazole (3.54 ng·L^−1^) and sulfadiazine (2.85 ng·L^−1^), with the rest of the antibiotics having mean concentrations below 1.7 ng·L^−1^.

Sulfonamides accounted for 45.47% of the antibiotics detected in the water samples, with a detection rate of 50%. The average concentration of sulfamethoxazole was 8.91 ng·L^−1^, which is much higher than that detected for any other sulfonamide. The proportion of macrolides was 32.34%, of which lincomycin hydrochloride had the highest detection rate of 93.75% with an average concentration of 8.36 ng·L^−1^. Quinolones accounted for 16.93% of detected antibiotics, of which ofloxacin had the highest detection rate of 68.75% with an average concentration of 5.08 ng·L^−1^. Norfloxacin, salafloxacin, lomefloxacin, fleroxacin and diflufenacin were all detected. This is due to the fact that most of the quinolone antibiotics have a strong adsorption capacity and are better able to adsorb sediment or suspended matter in rivers, making their detection rate low [24]. The proportion of tetracyclines was 5.26%, and their concentration in the sediment was relatively high due to the strong adsorption of hygromycin [25]. Chloramphenicol antibiotics were not detected at any of the eight sampling sites.

Comparing the Yellow River Estuary with other sections of the Yellow River Basin, a total of 14 antibiotics were detected in the Yellow River Estuary, as shown in Table 1, with concentrations starting from ND~415.53 ng·L^−1^ and the average concentration of the 34 antibiotics being 25.97 ng·L^−1^. In the Jinan section of the lower Yellow River [17], a total of 36 of the target antibiotics were detected in 35 sampling locations, and the concentrations of detected antibiotics starting from ND~13.462 ng·L^−1^, with an average concentration of 373.94 ng·L^−1^. Sulfonamides and macrolides were seen at a high rate; the total concentration of antibiotics seen in the Yellow River Delta section [18] during an abundant water period was ND~256.6 ng·L^−1^, with an average concentration of 15.09 ng·L^−1^; the total concentration of antibiotics detected in the intertidal zone of the Yellow River Delta [19] was ND~82.94 ng·L^−1^ with an average concentration of 10.37 ng·L^−1^; the total antibiotic concentration in surface waters such as canals, rivers and fish ponds in Kaifeng [20], a key city in the lower reaches of the Yellow River, Henan Province, was ND~12,224.99 ng·L^−1^, with an average concentration of 815.00 ng·L^−1^; meanwhile the total concentration of antibiotics in the Wei River [26], the largest tributary of the Yellow River Basin, was ND~573.26 ng·L^−1^ with an average concentration of 13.98 ng·L^−1^. In summary, the current level of antibiotic concentrations in the Yellow River Estuary is moderate.

### 3.2. Spatial Distribution of Antibiotics

**Figure 3:**
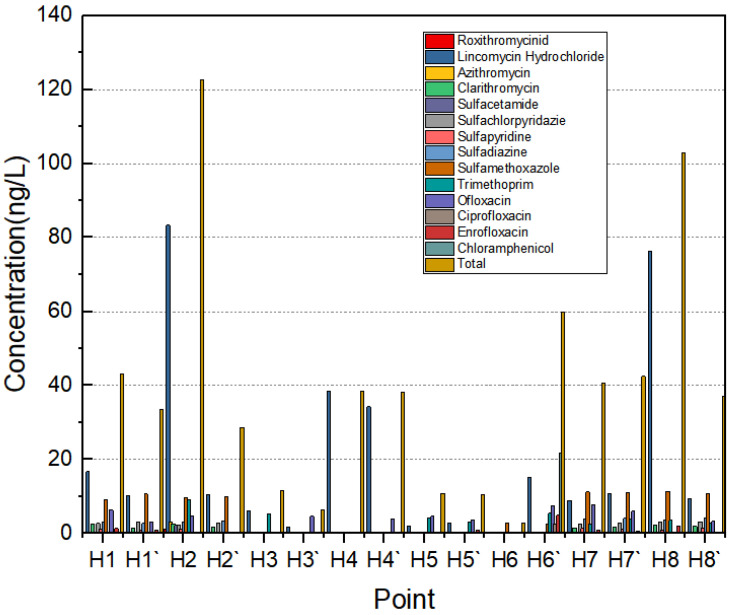
Distribution of antibiotic concentrations at the eight sampling sites.

The point distribution of antibiotics in the Yellow River Estuary is shown in Figure 3. As can be seen, the total number of antibiotics detected was highest at points H2 and H8 and lowest at points H3 and H5. Two substances were detected at all sampling locations, including one macrolide and one quinolone. Erythromycin, roxithromycin, telithromycin, sulfadimethoxypyrimidine, sulfathiazole, sulfamethoxazole, sulfamethoxypyrimidine, sulfadimethoxypyrimidine, sulfoquinoxaline, norfloxacin, salafloxacin, lomefloxacin, fleroxacin, difluoxacin, tetracycline, oxytetracycline, chlortetracycline, chloramphenicol, fluphenazole, methomycin and rifampicin were not detected at any of the eight sampling locations. Lincomycin hydrochloride had the highest detected mass concentration of 83.31 ng·L^−1^ at site H2 and doxycycline hydrochloride had the highest detected mass concentration of 21.86 ng·L^−1^ at site H6; sulfamethoxazole’s highest mass concentration was 11.32 ng·L^−1^, at site H8.

Overall, more than half of the eight sampling sites had higher concentrations of sulphonamide antibiotics than the other four categories, which are widely used in medical, agricultural, aquatic and livestock industries for the prevention and treatment of bacterial and protozoal infections because of their broad-spectrum antibacterial strength, low price, stable performance and sound therapeutic effects [26]. Sulfadiazine and sulfamethoxazole are commonly used to treat human diseases such as urinary tract infections and respiratory tract infections, and are the most widely used classes of sulfonamide antibiotics. Sulfamethoxazole is frequently used in farming to promote animal growth and increase production, while sulfadiazine is highly toxic. Sulfamethoxazole is quickly oxidized when exposed to light and is often used to suppress intestinal and soft skin tissue infections caused by sensitive bacteria, among other things. Sulphonamide antibiotics have a stable structure, degrade slowly in the environment and persist in the aqueous environment for an extended period of time. The sampling period coincided with the rainy season, with many cloudy days, which weakened natural degradation processes such as photodegradation, thus making the concentration of sulfonamide antibiotics significantly higher. Point H8 is the Lijin Hydrological Station, with a section width of 598 m. The main channel is 355 m wide and the beach area is 243 m wide. The beach is full of crops, which has a specific deterrent effect on the flooding of the beach. The Lijin Hydrological Station is part of the Yellow River Delta National Nature Reserve. Point H2 and point H3 are ecological tourist zones in the Yellow River Estuary. The general flow direction of rivers in China is from west to east; as point H2 is to the east of point H3, the water flows from point H3 to point H2, so the concentration of antibiotics at point H2 is higher than at point H3. Point H5 is the Feiyan Beach, which is one of the best areas in the Yellow River Estuary in terms of water quality due to the low level of human activities in the area.

### 3.3. Ecological Risk Assessment

**Figure 4:**
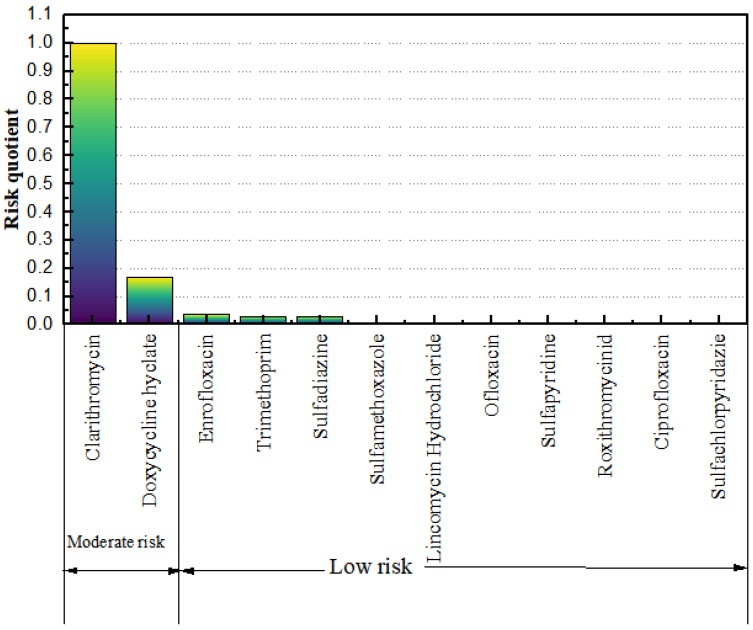
Risk quotient (RQ) values for antibiotics in water bodies of the Yellow River Estuary.

To better evaluate the risk level of antibiotics in the waters of the Yellow River Estuary, this study used the risk quotient method to perform a preliminary analysis of the 14 antibiotic-like substances detected. Toxicity data for each compound were screened from the literature, and the PNEC values (Table 2), as well as the risk quotient values (Figure 4), were calculated using the evaluation factor method. Of the 14 compounds detected, azithromycin and sulphonamide acetate were not evaluated here for ecological risk due to the lack of toxicity data from which to derive PNEC values.

## 4. Conclusions

The following conclusions were drawn from a survey of the pollution status and ecological risk assessment of 34 antibiotics at eight sites in the Yellow River Estuary. A total of 14 antibiotics were detected, with concentrations in the following descending order: sulphonamides, macrolides, quinolones, tetracyclines and chloramphenicol. The detection rate of sulfa antibiotics reached 45.47%, and the highest concentration detected at a single site was for lincomycin hydrochloride, with a concentration of 83.31 ng·L^−1^; the concentration levels of antibiotics at sites near villages, fishing grounds and hospitals were significantly higher than those around scenic areas, confirming that the concentrations of antibiotics in urban water bodies are closely related to human activities. The ecological risk assessment of the detected antibiotics using the risk quotient method showed that clarithromycin and doxycycline hydrochloride pose a medium risk, while lincomycin hydrochloride, sulfamethoxazole, meperidine, ofloxacin, enrofloxacin, sulfadiazine, roxithromycin, sulfapyridine, sulfadiazine and ciprofloxacin pose a low risk according to their concentrations in the water bodies of the Yellow River Estuary.

## Figures and Tables

**Table 1:** Comparison of antibiotic concentration levels in surface water in the Yellow River Estuary and other sections of the Yellow River Basin ^①^ ng·L^−1^.

Lakes (Year of Survey)	Antibiotic Concentration	Average Concentration
Yellow River Estuary (2022)	ND~415.53	25.97
Jinan section of the lower Yellow River (2022)	ND~13,462	373.94
Yellow River Delta Section (2019)	ND~256.6	15.09
Yellow River Delta intertidal zone (2016)	ND~82.94	10.37
Canal in Kaifeng, Henan, a key city on the lower reaches of the Yellow River (2022)	ND~12,224.99	815.00
Weihe River (2018)	ND~573.26	13.98

Note: ① ND stands for not detected.

**Table 2:** PNEC for common antibiotics ng·L^−1^.

Antibiotics	PNEC	References
Ofloxacin	21~17,400	[27,28,29]
Ciprofloxacin	2~30,000	[30,31]
Enrofloxacin	28.8~49	[32,33]
Roxithromycinid	4.3~10,000	[34]
Clarithromycin	2	[22,35]
Lincomycin Hydrochloride	50~50,000	[28,29,36]
Doxycycline hyclate	131	[37]
Sulfachlorpyridazie	2330~1,720,000	[38,39]
Sulfapyridine	460~5280	[38,39]
Sulfadiazine	107.394~135	[28,29,38,40]
Sulfamethoxazole	27~4674	[28,29,38,40]
Trimethoprim	29~255.516	[28,29,38,40]

## Data Availability

Not applicable.
